# Regulating Proteasome Activity

**DOI:** 10.3390/biom12030343

**Published:** 2022-02-23

**Authors:** Paolo Cascio, Gunnar Dittmar

**Affiliations:** 1 Department of Veterinary Sciences, University of Turin, Largo P. Braccini 2, Grugliasco, 10095 Turin, Italy; 2 Proteomics of Cellular Signaling, Department of Infection and Immunity, Luxembourg Institute of Health, 1a Rue Thomas Edison, L-1445 Strassen, Luxembourg; 3 Department of Life Sciences and Medicine, University of Luxembourg, Campus Belval, L-4367 Belvaux, Luxembourg

Strictly controlled degradation of the proteome is a key factor in maintaining cellular homeostasis and allows a rapid and effective response to a variety of different stress challenges. The central element of the regulatory degradative network that integrates different stimuli and signals is the proteasome, a macromolecular machine designed to selectively remove specific proteins according to the variable needs of the cell. Not surprisingly, given the extreme complexity and interdependence of the pathways involved, the proteolytic system has evolved with a modular organization based on a central hydrolytic element, the 20S proteasome, to which alternative regulatory modules can associate to fine-tune its activity. Perturbation of this delicate network is involved in the onset of various pathological conditions (e.g., cancer, neurodegenerative diseases, inflammatory and autoimmune disorders, infections), and in recent years strategies for modulating this proteolytic pathway (mainly through molecules that inhibit key enzymatic activities of this degradative system) have been shown to be extremely useful for both research and therapeutic purposes.

The scope of this Special Issue of *Biomolecules*, “Regulating Proteasome Activity”, was to provide a broad and updated overview of the main aspects of the properties and regulations of proteasomes, including functions and mechanisms of action of physiological proteasome interactors, as well as pharmacological molecules that inhibit its enzymatic activities. Ten manuscripts are published, namely seven reviews, one perspective study, and two original research articles that encompass several areas of proteasome research.

In their review, Alfred L. Goldberg and collaborators describe some striking, and only recently unveiled, mechanisms that allow for increased activation of the 26S proteasome and stimulation of protein degradation [1]. In fact, most proteasomes in cells are inactive, but upon binding an ubiquitinated substrate become activated by a two-step mechanism requiring association of the ubiquitin chain with Usp14 and the subsequent interaction of the loosely folded domain of the target protein with the ATPases of the proteasome. The initial activation step is signaled by the UBL domain in Usp14, and many UBL-domain-containing proteins (e.g., Rad23, Parkin) also activate the proteasome. Furthermore, ZFAND5 is a distinct type of activator that binds ubiquitin conjugates and the proteasome to stimulate proteolysis during muscle atrophy. Importantly, the proteasome’s activities are also regulated through subunit phosphorylation. Agents that increase levels of cAMP and activate PKA stimulate Rpn6 phosphorylation within minutes and enhance the selective degradation of short-lived proteins. Likewise, hormones, fasting, and exercise, which increase cAMP, all activate proteasomes and proteolysis in target tissues. Moreover, agents that increase cGMP and activate PKG also stimulate 26S activities but modify different subunit(s) and also stimulate the degradation of long-lived cell proteins. Of great interest, both kinases enhance the selective degradation of aggregation-prone proteins that cause neurodegenerative diseases. These new mechanisms regulating proteolysis, thoroughly described in the review, have clear physiological importance and therapeutic potential.

In their study, Krisztina Tar and coworkers [2] used a combination of yeast and human cell systems to investigate the role of Blm10/PA200 in the degradation of N-terminal Huntingtin fragments (N-Htt). In this way, they were able to demonstrate that human PA200 binds to N-Htt. The loss of Blm10 in yeast or PA200 in human cells results in increased mutant N-Htt aggregate formation and elevated cellular toxicity. Furthermore, Blm10 in vitro accelerates the proteasomal degradation of soluble N-Htt. Collectively, their data suggest that N-Htt is a new substrate for Blm10/PA200-proteasomes and point to new approaches in Huntington’s disease (HD) research.

By integrating molecular approaches with molecular docking studies Grazia Tundo, Diego Sbardella, Massimo Coletta and coworkers [3] have unveiled that the insulin-degrading enzyme (IDE), a ubiquitous and highly conserved Zn^2+^ peptidase often associated with proteasomes in cell-based models, is targeted in vitro by carfilzomib, a last generation proteasome inhibitor (PI) considered to be extremely specific in suppressing the chymotrypsin-like activity of the 20S proteosome. The drug behaves as a modulator of IDE activity, displaying an inhibitory effect that is over 10-fold lower than for the 20S proteasome. Notably, the interaction of IDE with the 20S enhances the inhibitory power of carfilzomib on proteasomes, so that the IDE-20S complex is an even better target of carfilzomib than the 20S alone in vitro. Nonetheless, IDE gene silencing after delivery of antisense oligonucleotides (siRNA) significantly reduced carfilzomib cytotoxicity in rMC1 cells, a validated model of Muller glia.

Proteasome inhibitors are essential tools for biomedical research. Three proteasome inhibitors, bortezomib, carfilzomib, and ixazomib, are approved by the FDA for treatment of multiple myeloma; another inhibitor, marizomib, is undergoing clinical trials. The proteolytic core of the proteasome has three pairs of active sites, β5, β2, and β1. All clinical inhibitors and inhibitors that are widely used as research tools inhibit multiple active sites. In the past decade, however, highly specific inhibitors of individual active sites and the distinct active sites of the lymphoid tissue-specific immunoproteasome have been developed. In his article [4], Alexei F. Kisselev provides a comprehensive overview of these site-specific inhibitors of mammalian proteasomes and describes their utilization in the studies on the biology of the active sites and their roles as drug targets for the treatment of different diseases.

A contribution by Stefanie Haberecht-Müller et al. [5] summarizes the function of the main proinflammatory cytokines and acute phase response proteins and their signaling pathways in inflammation-induced muscle atrophy with a focus on UPS-mediated protein degradation in muscle during sepsis. The regulation and target-specificity of the main E3 ubiquitin ligases (e.g., atrogin-1 and MuRF) in muscle atrophy and their mode of action on myofibrillar proteins is reported. Furthermore, the function of the standard- and immunoproteasome in inflammation-induced muscle atrophy is described and the effects of proteasome inhibitors as treatment strategies are discussed.

To circumvent the exposure to constant challenges such as pathogenic infections and commensal bacteria, epithelial and immune cells at the gut barrier require a rapid and efficient means to dynamically sense and respond to stimuli. The key to cellular and tissue proteostasis is the ubiquitin–proteasome system, which tightly regulates fundamental aspects of inflammatory signaling and protein quality control to provide rapid responses and protect from the accumulation of proteotoxic damage. In their review [6], Mohapatra, Eisenberg–Lerner and Merbl discuss proteasome-dependent regulation of the gut and highlight the pathophysiological consequences of the disarray of proteasomal control in the gut, in the context of aberrant inflammatory disorders and tumorigenesis.

Simranjot Bawa et al. [7] give a detailed account of human tripartite motif family of proteins 32 (TRIM32), a ubiquitous multifunctional protein that has demonstrated roles in differentiation, muscle physiology and regeneration, and tumor suppression. Mutations in TRIM32 result in two clinically diverse diseases. A mutation in the B-box domain gives rise to Bardet–Biedl syndrome (BBS), a disease whose clinical presentation involves no muscle pathology, while mutations in the NHL (NCL-1, HT2A, LIN-41) repeats of TRIM32 causes limb-girdle muscular dystrophy type 2H (LGMD2H). Interestingly, TRIM32 also functions as a tumor suppressor, but is paradoxically overexpressed in certain types of cancers. Moreover, recent evidence supports a role for TRIM32 in glycolytic-mediated cell growth, thus providing a possible mechanism for TRIM32 in the accumulation of cellular biomass during regeneration and tumorigenesis. Furthermore, the review delves into the utility of the Drosophila model, a unique system to study glycolysis and anabolic pathways that contribute to the growth and homeostasis of both normal and tumor tissues.

PA28 (also known as 11S, REG or PSME) is a family of proteasome regulators. In jawed vertebrates they are represented by three paralogs, PA28α, PA28β, and PA28γ, which assemble as heptameric hetero (PA28αβ) or homo (PA28γ) rings on one or both extremities of the 20S proteasome cylindrical structure, or at the free end of asymmetric, singly capped 26S particles to form so-called hybrid proteasomes. In his review, Paolo Cascio details the molecular mechanisms and biochemical properties of PA28γ, which are likely to account for its various and complex biological functions and highlight the common features with the PA28αβ paralog [8]. In fact, PA28α and PA28β seem to have appeared more recently and to have evolved very rapidly to perform new functions that are specifically aimed at optimizing the process of MHC class I antigen presentation. On the contrary, PA28γ seems to be a slow-evolving gene that is most similar to the common ancestor of the family of PA28 activators, and very likely retains its original functions [9]. The article emphasizes how most of the diverse cellular functions of PA28γ (e.g., regulation of cell growth and proliferation, apoptosis, chromatin structure and organization, response to DNA damage) seem to depend on its ability to markedly enhance degradation rates of regulatory protein by the 20S proteasome. Moreover, the results of recent studies showing that PA28γ endows the 20S proteasome with the ability to very efficiently degrade unfolded proteins [10] are presented and discussed in light of the known biological properties of the activator.

The article by Indrajit Sahu and Michael H. Glickman [11] reviews recent advances in our understanding of the biochemical and structural features that underlie the proteolytic mechanism of free 20S proteasomes, which are abundant in many eukaryotic cells, and which can degrade substrates with considerable unstructured stretches. The two outer α-rings of 20S proteasomes provide a number of potential docking sites for loosely folded polypeptides. The binding of a substrate can induce asymmetric conformational changes, trigger gate opening, and initiate its own degradation through a protease-driven translocation mechanism. Consequently, the substrate translocates through two additional narrow apertures augmented by the β-catalytic active sites. The overall pulling force through the two annuli results in a protease-like unfolding of the substrate and subsequent proteolysis in the catalytic chamber.

Finally, Marta L. Mendes and Gunnar Dittmar [12] describe how cross-linking mass spectrometry (CLMS) is now routinely used in integrative structural biology studies, and it promises to take integrative structural biology to the next level, answering some of the questions about the structure of the 19S regulatory particle and on the different conformational states of the 26S proteasome that have not yet found a definitive answer through cryo-electron microscopy (cryo-EM).

To conclude, this Special Issue of *Biomolecules* describes important findings relating to the role of proteasomes in physiological and pathological conditions and their involvement in metabolic regulation. The function of different modulators (both inhibitors and activators) has been extensively described, highlighting their molecular mechanisms as well as their biological and cellular implications. All these data can be extremely useful to understand the role of the proteasome in the various cellular processes in greater detail and to develop pharmacological strategies aimed at modulating its activity for therapeutic and clinical purposes. 

## References

[1] Goldberg A.L., Kim H.T., Lee D., Collins G.A. (2021). Mechanisms That Activate 26S Proteasomes and Enhance Protein Degradation. Biomolecules.

[2] Aladdin A., Yao Y., Yang C., Kahlert G., Ghani M., Király N., Boratkó A., Uray K., Dittmar G., Tar K. (2020). The Proteasome Activators Blm10/PA200 Enhance the Proteasomal Degradation of N-Terminal Huntingtin. Biomolecules.

[3] Raffaella T.G., Diego S., Francesco O., Giuseppe G., Stefano M., Grazia A.M., Maria S.A., Danilo M., Francesco B., Gabriele M. (2022). Insulin-Degrading Enzyme Is a Non Proteasomal Target of Carfilzomib and Affects the 20S Proteasome Inhibition by the Drug. Biomolecules.

[4] Kisselev A.F. (2021). Site-Specific Proteasome Inhibitors. Biomolecules.

[5] Haberecht-Müller S., Krüger E., Fielitz J. (2021). Out of Control: The Role of the Ubiquitin Proteasome System in Skeletal Muscle during Inflammation. Biomolecules.

[6] Mohapatra G., Eisenberg-Lerner A., Merbl Y. (2021). Gatekeepers of the Gut: The Roles of Proteasomes at the Gastrointestinal Barrier. Biomolecules.

[7] Bawa S., Piccirillo R., Geisbrecht E.R. (2021). TRIM32: A Multifunctional Protein Involved in Muscle Homeostasis, Glucose Metabolism, and Tumorigenesis. Biomolecules.

[8] Cascio P. (2021). PA28γ: New Insights on an Ancient Proteasome Activator. Biomolecules.

[9] Fort P., Kajava A.V., Delsuc F., Coux O. (2015). Evolution of Proteasome Regulators in Eukaryotes. Genome Biol. Evol..

[10] Frayssinhes J., Cerruti F., Laulin J., Cattaneo A., Bachi A., Apcher S., Coux O., Cascio P. (2022). PA28 gamma-20S proteasome is a proteolytic complex committed to degrade unfolded proteins. Cell. Mol. Life Sci..

[11] Sahu I., Glickman M.H. (2021). Structural Insights into Substrate Recognition and Processing by the 20S Proteasome. Biomolecules.

[12] Mendes M.L., Dittmar G. (2021). Analysis of the Dynamic Proteasome Structure by Cross-Linking Mass Spectrometry. Biomolecules.

